# Current Perceptions of Sustainable Diets in Ireland and the Outlook of Circular Eating Practices

**DOI:** 10.3390/foods12214003

**Published:** 2023-11-01

**Authors:** Shelley Fox, Francesco Noci, Owen Kenny, Maria Dermiki

**Affiliations:** 1 Department of Health and Nutritional Sciences, Faculty of Science, Atlantic Technological University, F91 YW50 Sligo, Ireland; shelley.fox@research.atu.ie (S.F.); owen.kenny@atu.ie (O.K.); 2 Department of Sports Exercise and Nutrition, School of Science and Computing, Atlantic Technological University, H91 T8NW Galway, Ireland; francesco.noci@atu.ie

**Keywords:** agricultural by-products, qualitative research, food waste, food choice, changing habits

## Abstract

Encouraging sustainable dietary practices is a positive step towards alleviating the negative impact of increasing food waste. This study explores consumers’ perceptions of sustainable diets and factors influencing their adoption of sustainable practices, such as circular eating. Fifteen consumers in Ireland aged 18–65+ years were recruited for a qualitative study exploring their views on sustainable diets and their opinions on the use of peels, trimmings, and other by-products from fruit and vegetables as potential new ingredients or new circular food products. Age, gender, dietary preferences and educational background were important considerations during recruitment. Online interviews were conducted, and the data were analyzed using thematic analysis managed by NVivo 12. The results highlighted two overarching themes impacting circular eating, namely, food choice motives and awareness of food waste. These were linked to the participants’ knowledge of and potential adoption of sustainable diets. Daily trade-offs were evident, linked to the product and the person who was also strongly influenced by their micro-environment. Consumer education from credible sources would be required to create awareness of circular eating. Most respondents were positively disposed to the concept of circular eating, as long as food safety concerns and affordability considerations were addressed through industry transparency.

## 1. Introduction

Globally, a quarter of all food produced does not reach our tables [1,2]. In Ireland, currently, 1 million tons of food are wasted annually [3]. Food loss and waste can be generated at each stage along the supply chain, with losses occurring at primary production, post-harvesting, and processing stages, while food waste occurs at retail, distribution, and consumer stages [4]. Food waste specifically contributes up to 60% of the average household’s general waste annually, indicating a considerable issue to be addressed [5]. A factor that contributes to food losses is food surplus, which is food that is produced in excess to meet potential orders that may never be realized. This is further threatening food security and generating potentially avoidable food waste [6]. The impact of excessive greenhouse gas emissions generated by food waste disposed to landfills has resulted in discussion and action plans focused on food waste reduction [7,8]. The United Nations’ sustainable development goals (UN SDG), specifically SDG 12, aim to improve production and consumption patterns, include targets addressing food waste, and encourage sustainable consumption [8]. 

The circular economy is a concept that has been embraced at the European level to encourage improved sustainable consumption. Frameworks have been developed focusing on moving from the traditional linear models to more sustainable models [9,10,11]. However, this is a complex change for the food supply chain. Moving from large-scale industrial operations to smaller networked circular systems [12] requires implementation not only within individual businesses but also across stakeholders, creating interconnectedness, transparency, and greater opportunity for success [13]. Even though the level of food waste generated from current food systems is unsustainable, linear systems are efficient in terms of yields, quality, and safety, moving to a more circular system may create new food safety risks [14]. 

One such circular system that focuses on reducing waste and achieving sustainable consumption is the revalorization of food waste [6]. This has been referred to as “Circular Eating”, “upcycled foods”, or “waste-to-value” as it reuses as much of the surplus material as possible aiming to avoid the generation of waste and creating a new food ingredient or finished product [15,16,17,18,19]. However, consumer acceptance of these products and ingredients is essential for their uptake. Aschemann-Witzel and Stangherlin [20], in their review, found that consumer, product, and situation-related factors affected the acceptance of ‘upcycled’ foods. These are the factors influencing general food choice motives as described by Köster [21]. Previous studies have explored the impact of food choice motives on consumer acceptance of different dietary practices [22,23,24,25,26] using the theoretical model on food choice motives developed by Steptoe et al. sometimes adapted to suit the studies [27]. Verain et al. [28] established and validated a sustainability questionnaire that compliments Steptoe’s food choice questionnaire for sustainability-related studies. Although questionnaires have been validated to measure food choice motives and potentially green consumer behavior [29], the concept of circular eating has not been studied extensively and is a relatively new social phenomenon that calls for the use of qualitative research methodologies to provide rich data [30]. 

This research focuses on the revalorization of by-products generated from fruit and vegetable processing since one-third of all food waste worldwide comes from the horticulture sector [4]. Specifically in Ireland, 21% of food waste and losses are generated from horticulture [31]. To explore consumers’ acceptance of circular eating, first, it is important to understand the nuances around why consumers choose the foods they choose, and since the concept is considered a sustainable consumption practice, it is also important to understand consumers’ perceptions of sustainability. Although sustainability is divided into social, environmental, and economic pillars, previous research has shown that consumer’s understanding usually focuses on environmental issues [32]. When exploring factors affecting sustainable dietary practices, the most important ones were consumer-related factors such as their sociodemographic characteristics and their micro- and macro-environment [18,33,34], similar to the ones affecting their general food choice motives. 

Aschemann-Witezel and Stangherlin’s review on the factors affecting acceptance of upcycled material [20] noted an absence of qualitative research in this field, suggesting that “qualitative studies might show diverse associations to the waste-to-value products, allowing studies to identify which aspects consumers consider positive or negative”. The current qualitative study aims to identify consumers’ willingness to accept the use of surplus material generated from the fruit and vegetable sector, which is normally sent to the waste stream, such as peel, trimmings, and other by-products, in new sustainable food products. Based on background information, the specific objectives of this study are presented:

- To explore the perception of sustainability and sustainable diets
- To understand general food choice motives
- To explore consumer understanding of food waste
- To explore the factors affecting the acceptance of the use of peels, trimmings, or by-products from fruit and vegetables in new foods

To satisfy the aim and objectives of this study, two main research questions were employed: (Q1) How do Irish consumers perceive sustainable diets, and (Q2) Which factors affect their willingness to consume food products containing peels, trimmings, or by-products generated during fruit and vegetable processing. Furthermore, to gain a deeper understanding of the topic, the two main research questions were divided into four embedded questions: (Q1a) What are the factors related to the sociodemographic characteristics of participants that affect their perception towards sustainable diets (and consumption of products containing peels, trimmings, or by-products), (Q1b) What are the characteristics of the diets that make them sustainable according to the participants, (Q2a) What is the consumer’s understanding of food waste, circular eating, and by-products from the food industry, and (Q2b) How do consumers perceive food products containing by-products generated from the fruit and vegetable sector. 

## 2. Materials and Methods

### 2.1. Materials and Methods

**Figure 1:**
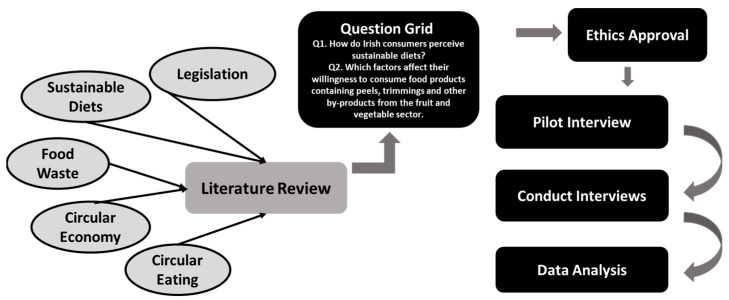
Flow chart describing the generation of the research questions and the steps followed to answer them.

To answer the research questions, an interpretivist paradigm was employed, and an exploratory qualitative research design was selected. Qualitative research allows the researcher to understand the subjective experiences of the participants [35] by putting the researcher in connection with the participants. Figure 1 shows the flow of how the research questions were guided by the literature and the steps of data collection and analysis. This study was approved by the Research Ethics Committee of the Institute of Technology Sligo, Ireland, in May 2021 (IREC Reference No: 2020048). 

### 2.2. Sampling Procedure and Recruitment

**Figure 2:**
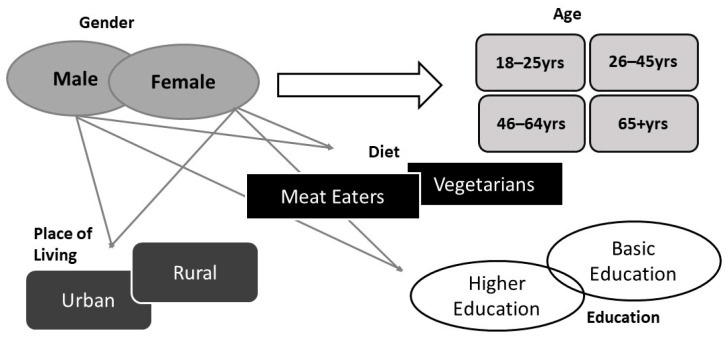
Sample triangulation to achieve reliability in the research design.

Convenience purposeful sampling was adopted to recruit consumers for this study who met the sample triangulation criteria (Figure 2), by advertising on social media and emailing the students and staff of Atlantic Technological University (Sligo and Galway campuses) via gatekeepers. This triangulation identifies age and gender as two key sociodemographic characteristics as Culliford and Bradbury [34] showed that there is an interaction between gender and age in the readiness to adopt sustainable dietary behaviors. It was also important to include current dietary behaviors as a criterion as vegetarians may have a different outlook to meat eaters in relation to sustainable diets. The other criteria selected for this study were the locations that the respondents live in, urban or rural, and educational background, as Barone et al. [33] showed an association between age, education, and the locations that respondents live in terms of knowledge around sustainable diets. Snowball sampling was also employed to recruit additional participants [36]. 

### 2.3. Interview Procedure

A question grid was developed using insight from the literature as seen in Figure 1 (see also Section S1 Supplementary Material for more details). The interview was divided into three sections. Section one was a short survey to capture demographic information specified in sample triangulation (Figure 2). This information was captured using Qualtrics XM (first release 2005, copyright year 2021, the US, available at https://www.qualtrics.com (accessed on 10 June 2021)). Section two focused on food choice motives and sustainable diets. Section three focused on food waste management and circular eating. The interviews were arranged via video-conferencing software MS Teams (version 1.5.00.9163) or Zoom (version 5.6.7(1016)) and were conducted between June and September 2021. They were concluded when data saturation was achieved, as there was no more new information coming through [36]. The duration of each interview was between 20 and 40 min. The recorded interviews were transcribed verbatim using Otter transcription (version 3.30.0-90c819b7, US, available at https://otter.ai (accessed on 22 June 2021)). To ensure accuracy, the interviews were listened back on three separate occasions, and the transcripts were amended as required. This resulted in 167 pages of transcripts for analysis.

### 2.4. Participants

A total of fifteen consumers (older than 18 years old, residing in Ireland) were interviewed, and their socio-demographic characteristics can be seen in Table 1. This information was collected to understand how sociodemographic characteristics might influence the consumer’s motives around sustainable diets and food waste management. Pseudonyms were assigned to each participant to preserve anonymity and are used in the results section along with key sociodemographic information, where quotations are displayed. 

### 2.5. Data Analysis

The methodology applied for data analysis was Reflexive Thematic Analysis [37,38]. A hybrid approach was employed using deductive and inductive reasoning when analyzing the data with some potential themes identified in the literature in advance of commencing the coding process [39]. Each transcript was read and reviewed multiple times, important text was highlighted manually, and notes were recorded (Phase one). NVivo 12 software (Burlington, VT, USA) was used to manage the six stages of coding and data analysis. Phase two was often referred to as open coding, where important phrases or sentences are assigned descriptive codes. At the end of phase two, there were 385 loose codes. Phase three focused on generating initial themes or categories from coded and collated data. Memos were generated in NVivo to explain the researcher’s analysis during this process, and these were linked to the categories. When working on this stage of the analysis, it was also evident that links existed between themes. This occurred within and across both research questions. Phase four reviewed the initial themes and developed these further. At this point, some of the initial themes became subthemes of larger over-arching themes. By delving into this further, a more cohesive coding structure was developed. Phase five focused on refining and defining the final themes and identifying any connections between themes and subthemes. Concept maps and exploratory diagrams in NVivo allowed the researcher to visualize the data and identify coding similarities. When analyzing the data, the researcher also looked at the sociodemographic information of the participants, collected using the Qualtrics survey, and examined how these sociodemographic characteristics impacted the main themes and subthemes. Phase six was the writing-up stage where the themes and subthemes were reviewed to identify how they relate to each other and how they answer the research questions. NVivo visualization tools were used to help explain these themes (see Section S2 Supplementary Material for examples), some of which are presented in the results and discussion section. 

## 3. Results and Discussion

### 3.1. Overview of Main Themes

**Figure 3:**
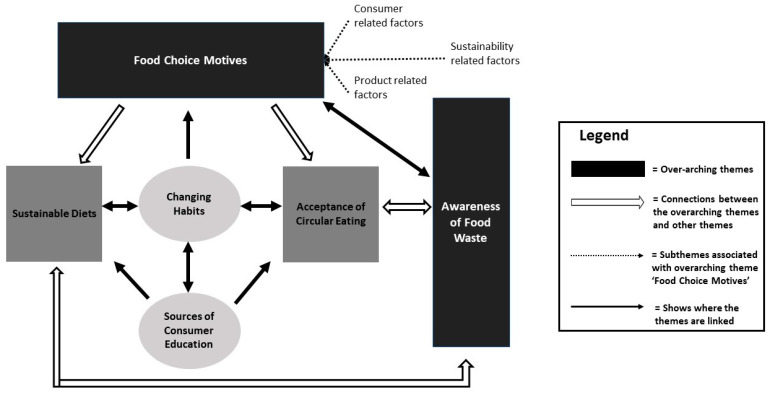
Overview of main themes identified in this study and their interconnection.

Thematic analysis of the data generated from the interviews identified six main themes. These themes and associated subthemes along with the frequency that they were mentioned by the participants can be seen in Table 2. Figure 3 represents the connection between the six themes. Food choice motives and awareness of food waste were two overarching themes that informed consumers’ beliefs relating to sustainable diets and acceptance of circular eating (in bold in Table 2) and were directly linked to the two main research questions. The sustainable diet theme was the most frequently referenced (see Table 2), most likely due to the interview questions, which focused heavily on sustainability, sustainable foods, and sustainable diets.

### 3.2. Concept of Sustainability, Sustainable Foods, and Sustainable Diets

#### 3.2.1. Awareness of Sustainability

**Figure 4:**
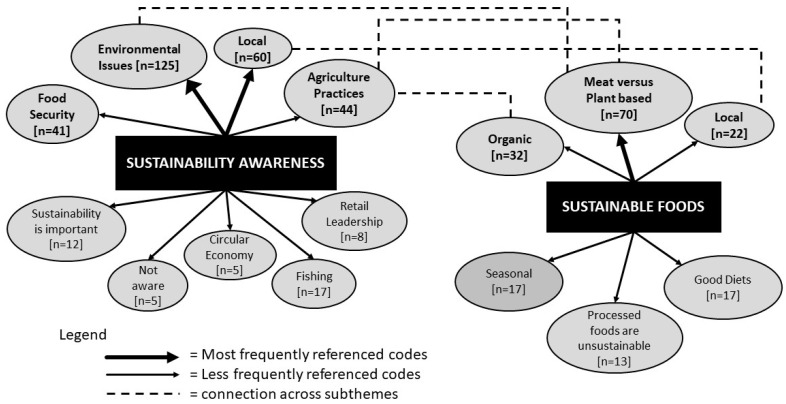
Schematic of codes associated with the subtheme’s sustainability awareness and sustainable foods (codes with higher frequencies are in bold and larger font).

To address the first research question, ‘How do Irish consumers perceive sustainable diets?’, it was imperative to gain an understanding of how they viewed sustainability in general. This is how the subtheme on consumer’s awareness of sustainability was derived (Table 2). The results highlighted a lack of educated knowledge about sustainability or sustainable foods (subtheme in Table 2) with dietary information mostly sourced online (theme on sources of consumer education in Table 2). Similar to other studies [32], the participants struggled to discuss this topic initially with the resulting discussion focusing mostly on environmental issues (shown in Figure 4) such as the strain on natural resources, food miles, biodiversity loss, and excessive use of plastic packaging. Biodiversity loss and unsustainable farming practices were mentioned, particularly in relation to the beef sector, as ***Mark, 26–45 yrs, a postgraduate student in environmental science*** said, *“There’s no chance for nature to work in harmony with it, it just works against it and is just totally unsustainable”*. However, agriculture is a very important sector for Ireland, with €15.4 billion worth of agri-food exports, 2.4 billion EUR of these comes from the beef sector, as noted in the Department of Agriculture, Food, and Marine (DAFM) annual review for 2022 [40]. DAFM also confirmed there are more than 170 thousand people employed in the agrifood sector, which may cause some resistance to suggested changes. As this is an important industry for Ireland, the Ag Climatise roadmap has been developed to assist the farming community in making sustainable changes to meet climate targets and protect their livelihoods [41]. This further feeds into the EU Commission’s common agricultural policy (CAP), which combines social, economic, and environmental approaches to achieve sustainable practices in agriculture within the EU member states [42].

Consumers’ strong association between environmental issues and sustainability has been found in numerous studies previously [5,32,34,43]. However, over the course of the discussions, the participants of this present study broadly acknowledged the three pillars of sustainability and mentioned most of the factors listed in the FAO’s 2010 [44] definition of a sustainable diet showing an unconscious awareness. Outside of the environmental issues, ‘local’ was almost the first word spoken by the participants, such as locally produced, sourcing from local markets, this factored in Irish produce, too, due to the size and scale of the country (Figure 4).

The participants were also quite concerned about food security and expressed a need for food to be available for future generations. In addition to food security, animal welfare was one of the most common subthemes. For this reason, some have chosen a vegetarian or vegan lifestyle. Leading on from this, there was also a concern in relation to mass food production, whether it is animal production and processing or the production of cheap, highly processed foods. These were viewed negatively overall and impacted their food choices. However, when considering the social pillar of sustainability, a balance is required. Particularly, as the agrifood sector offers much-needed employment, which is of particular importance in rural areas [45]. 

There was a realisation among some of the consumers interviewed that in Western countries where people were privileged, in terms of easy access to fresh produce. Participants believed that retailers should take a leading role in improving the availability of sustainable products and marketing of products, so consumers have more information available to make informed choices. However, ***Nina, 26–45 yrs, Dietician*** and ***Ben, 18–25 yrs, a Nutrition student*** mentioned *“greenwashing”* and how they have a certain level of distrust in the messaging used to promote sustainability. This scepticism was also identified in a recent review of consumers’ perception of sustainability related to foods [32]. The issue of greenwashing has been recognised by the EU Commission recently, and a proposal for a directive on green claims has been made to stop companies from making misleading claims [46]. 

Some participants identified the circular economy or some form of recycling as a way of achieving sustainability. However, overall, it was acknowledged that “*as a society we need to be kinder to the planet and embrace sustainability*” and embracing the circular economy is an important first step. 

#### 3.2.2. Sustainable Foods and Sustainable Diets

When the participants were asked to mention which foods were sustainable (subtheme of *sustainable foods* in Table 2), the focus of the discussion was centred on *meat versus plant-based* (n = 70) as shown in Figure 4. Most participants, even those following a predominantly meat-based diet, believed they should be reducing their meat intake and incorporating at least one meat-free day per week. This is most likely due to prominent media campaigns such as ‘Veganuary’ or ‘Meat Free Monday’, which encourage consumers to make small changes in their current diet and select plant-based alternatives [47,48]. This view is in line with recommendations from Willet et al. [49] in the EAT Lancet report, which stated that to become more sustainable, diets should be less reliant on animal products. The suggested recommendations may be quite difficult for some Irish consumers due to the strong agrifood culture present in Ireland [40], along with the long-standing traditional dietary practice of consuming meat, potatoes, and vegetables as their main meal. This is evident in many countries where meat consumption is attached to family or social occasions and other religious and cultural events [50]. Some participants were also sceptical and felt that the ‘sustainable plant-based diet’ was more of a trend or a marketing tagline and that a balanced diet would be healthier. Van Bussel et al. [32] review paper suggested that sustainable food consumption is considered an added bonus to a healthy diet rather than the main dietary consideration. A Teagasc report by McCarthy [51] recommended a more holistic approach rather than only focusing on reducing meat intake, stating that a suite of measures would be required. This highlights the potentially conflicting messaging around the best way to achieve a sustainable diet. 

Organic produce, the second most popular subtheme in relation to sustainable foods, was viewed as “*kinder to the land with no chemicals or pesticides used during processing*”, confirming the participants’ beliefs about the sustainability of current agricultural practices. Other studies have also found an association between organic food and an environmentally friendly meal [32,52]. However, Redlichova et al. [53] suggested that organic farming requires more land and uses more energy and therefore may not be as sustainable as consumers often believe. Local produce was suggested to reduce air miles and lower carbon footprint. It also supports local producers, who were viewed by the participants as important, contributing to the social pillar of sustainability. A number of those interviewed mentioned an online farmers market called ‘neighbour foods’, which sources produce from producers in a local area [48]. Fresh, natural, and seasonal were suggested as important in terms of sustainable produce. However, it was viewed that it would be easier to eat seasonally during the summer and autumn months when local produce was readily available. It is also important to note that Ireland imports a considerable amount of fruit and vegetables annually, not only exotic fruit that cannot be grown in Ireland; for example, 66,000 tonnes of apples and 75,000 tonnes of potatoes were imported in 2020 [54]. 

When asked to define sustainable diets, more than half of those interviewed defined sustainable diets as balanced, natural, and healthy, where dietary choices are based on the food pyramid [55]. This agrees with some elements of the FAO’s 2010 definition of a sustainable diet [44], as “*a diet with low environmental impacts, contributes to food and nutrition security and to a healthy life for present and future generations. Sustainable diets are protective and respectful of biodiversity and ecosystem, culturally acceptable, economically fair and affordable, nutritionally adequate, safe and healthy, while optimizing natural and human resources*”. While fruit and vegetables were considered to contribute to a more sustainable diet, processed foods were considered unsustainable by under half of those interviewed. The participants also commented on the amount of waste generated within the big industry to produce small speciality foods. The level of additives in processed foods was also a concern for participants, feeding into a level of distrust towards to food industry. ***Michelle 26–45 yrs, an organic farmer and part time food industry auditor*** thinks *“the processing industry has a lot to come to terms with regarding our diets now, there’s a generation who do not know how to cook and they go for the easy option”*. However, it is also important to note that processing technologies developed over many decades have also promoted food safety and preservation, as well as formulating nutritious, affordable foods that are appealing to consumers [56]. This highlights the potential disconnect between the consumer and the food industry and the need for education with consumers to help them understand the different types of food processing.

### 3.3. Food Choice Motives Affecting Adoption of Sustainable Diets

Food choice motives were an overarching theme guiding sustainable food choices and circular eating (Figure 3). It had a clear impact on whether participants would adopt a sustainable diet or under which conditions they would accept circular eating. These food choice motives were mostly consumer- or product-related factors, with sustainability factors also evident but to a lesser extent (Table 3).

#### 3.3.1. Consumer-Related Factors

Within consumer-related factors (Table 3), health considerations were one of the most important factors as revealed by the frequency of the codes associated with health considerations. These considerations were focused on maintaining a balanced diet, having awareness of the nutritional quality of their food, and ensuring they followed dietary guidelines such as the food pyramid [55]. Culliford and Bradbury [34] also found health to be an important food choice motive for UK consumers and noted that sustainable diets are suggested to improve health, by reducing the volume of red meat intake, which in turn may help in the reduction of the risk of cardiovascular diseases and dietary-related cancers [57]. Therefore, by introducing more plant-based foods into their current diets, consumer health may also see positive changes. Dietary guidelines, such as the UK’s Eat Well Guide [58] and Healthy Ireland [38], although advocating to include more grains and legumes, do allow for some meat in the diet, which reinforces the concept of the balanced diet as do other publications such as Willet et al., Eat Lancet report [49]. 

Eating habits (Table 3) influenced by tradition were discussed frequently with personal preferences often dictating the foods consumed. As ***Anna (aged 26–45 yrs, family of 4 (2 adults, 2 children)*** explained “*I think traditionally it would have been habit, you know, we’re both from very traditional families where you have your meat, veg and potatoes”*. The micro-environment of those interviewed was shown to have a major impact on their food choice motives, particularly the influence of family members, for example, including a vegetarian meal for the visiting relative or the consideration of children’s nutritional needs. As most participants in this study were aged 26–45 years (n = 11) and came from households with children in the home, this impacted their food choices and consequently the adoption of sustainable diets. Although the participants broadly acknowledged the benefits of moving to a more sustainable diet, they identified barriers such as a lack of knowledge of sustainable foods or vegetarian recipes; they also highlighted their belief that sustainable diets were more expensive, and trade-offs would be inevitable. These barriers often outweighed the benefits of making this change. 

Socio-demographic factors connected to knowledge of nutrition gained through employment and educational background affected food choices. Moreover, the place of residents impacted participants’ access to food retailers, which in turn, impacted choices, forcing them to shop smarter. For example, those in rural locations were forced to buy in bulk compared to the ones living in urban areas. Gender is one of the consumer-related factors affecting food choices, and several studies have identified gender effects, noting females were more open to adopting plant-based diets [18,33,34]. However, as this study was an exploratory qualitative study with fewer participants, this was not evident. In fact, the two vegan participants were both male, and all other participants across the age brackets 18–46 years were already incorporating or planning to incorporate at least one vegan or vegetarian main meal into their normal weekly diet. Age was mentioned by two participants as a barrier to change, linked to traditional dietary habits. Age could impact the living situation, for example, living with children or alone, which in turn affects food choices. De Visser et al. [59] found younger participants who had already stopped eating meat saw mostly benefits rather than barriers to adopting a vegetarian diet, and they also displayed a stronger negative attitude towards meat production and believed it was not difficult to remove meat from their diet. This was perhaps because this cohort did not have to consider young children’s dietary needs, which impacted the food choice motives of several of the 26–45-year-old participants in this study. The older participants in this study (male aged 64 years and female aged 66 years) were less inclined to incorporate plant-based foods in their diet. Lea et al. [60] suggested older adults were less interested in changing their dietary practices. Grasso et al. [61] found ‘fussiness’ to be a factor in the acceptance of plant-based foods with older participants. Based on the results from this present study and the other studies mentioned above, targeted campaigns aimed at older demographics may be useful in encouraging the uptake of sustainable diets within this cohort. 

#### 3.3.2. Product-Related Factors

**Figure 5:**
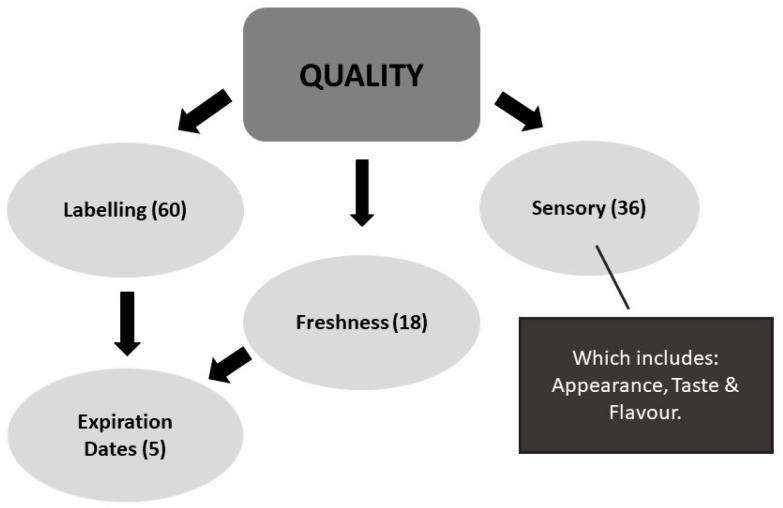
Breakdown of the quality factors related to the product, which impacted participants’ food choice motives (the frequencies are included in (brackets) beside the factors).

Factors related to products heavily influenced the participant’s food choice motives. Product quality was of the utmost importance and was mentioned by most participants, followed closely by economic considerations such as affordability. Convenience was discussed mostly in terms of availability and variety from the various retailers. While the term quality in the food industry comes under the remit of quality assurance, for the participants, several codes were associated with quality as seen in Figure 5. For example, labelling was discussed most often, as the way most participants sourced information on ingredients, nutritional information, and shelf life of products. Sensorial factors such as appearance, taste, and flavour were also important to those interviewed. Freshness was used in combination with appearance and other sensorial factors when making purchase decisions. The discussion around expiration dates was two-fold, with participants ensuring that they purchase produce with the longest shelf life, so they do not have to visit the supermarket outside of the weekly shop. However, some of the participants used a more liberal approach such as *“the sniff test”* to avoid discarding food that is still acceptable even though the best-before date has expired. This demonstrates a more sustainable approach to managing food in the home setting and helps to reduce food waste. Food labelling legislation, specifically date marking, is currently undergoing a review, in line with recommendations contained in the EU farm-to-fork strategy [7], where the overall aim is to deliver expiration dates in a more consumer-friendly manner and reduce confusion, which in turn should help to reduce unnecessary food waste [62].

#### 3.3.3. Sustainability Factors 

Sustainability factors were another theme related to food choices that were usually negated by other food choice motives such as cost and maintaining a balanced diet. Although most participants identified ‘local’ as the first word that came to mind when thinking about sustainable foods, they purchased products predominantly from the major retailers located in Ireland, rather than local markets. As most of the participants live in rural locations, making a weekly trip to purchase their groceries is the most convenient option for them, fitting in with their busy lives. Discount retailers were mentioned frequently in terms of affordability for those on reduced incomes. One-third of the participants listed their beliefs in relation to the environment such as excessive usage of natural resources and the extent of plastic packaging on produce as impacting their food choice motives, linking this back to trying to maintain a balanced diet and source local produce. ***Michelle, aged 26–45 yrs, a vegetarian and organic farmer***, noted that *“If you eat a balanced diet and you know where the fruit, veg and meat comes from, you’re going to be much healthier and the environment around you is going to be much healthier too”*. Food choice motives associated with sustainability were also evident when participants were describing sustainable foods and sustainable diets, highlighting how the theme of food choices was an overarching theme.

Overall, when considering food choice motives, the main outcome was that participants tried to ensure a healthy, nutritionally balanced diet, whilst maintaining traditional habits and affordability within the constraints of their individual micro-environment. These findings were in agreement with the review by Van Bussel et al., 2022, who found that sustainability does not ‘yet’ influence consumers’ food choices, with price, taste, and health more relevant [32]. Sustainability factors such as environmental or social issues were mentioned in this study but were of limited importance as they were mentioned less frequently. These factors impacting the adoption of sustainable dietary practices reflect the theory of planned behaviour, highlighting the influence of the subjective norm and perceived behavioural control on the intention to change behaviours [63]. In this study, the subjective norms are often linked to personal preference and traditional habits, although awareness of the need to move to more sustainable dietary practices is evident, these subjective norms impact the intention to change. 

### 3.4. Changing Habits 

Traditional habits are ingrained and often handed down from generation to generation, making them very difficult to change. However, it was recognised by the participants that it was necessary to change these habits to move to more sustainable choices. This theme was recognised as an interconnecting theme between sustainable diets and circular eating (Figure 3).

In terms of sustainability factors linked to changing habits, there is an impending fear that time is running out to achieve any real success and change the trajectory of the climate crisis. This study focused on two key areas closely linked to sustainable production and consumption, which is also the focus of SDG12. It not only identified areas for improvement to aid consumers in making informed decisions but also highlighted barriers that need to be addressed before change could happen. There are ingrained dietary practices such as traditional daily meat consumption that inform current food choice motives that may impede the transition towards the adoption of a more sustainable diet. Participants in the older age categories, such as Rosemary, aged 66 years, retired with a background in environmental science, noted they were very settled in their dietary patterns, which has meat as a mainstay, and would not be inclined to change habits, indicating a potential effect of age on the adoption of sustainable diets as mentioned earlier and was also found by Grasso et al. [61]. *“You can put it as lack of imagination, unwillingness to engage in serious cooking. I also believe that having protein is very important. I see meat as pre-packaged easy protein”*. Similar habits were identified in other studies, where the strong attachment to meat was attributed to taste and familiarity, as well as a source of key nutrients, which consumers were confident enough to prepare due to experience [34,52].

This study identified two main subthemes within the changing habit theme, firstly changing eating behaviors and secondly changing shopping behaviors. Both require the individual to make changes within their micro-environment. This was identified as a factor in their food choice motives, demonstrating the dilemmas consumers face, which often leads to avoidance of making changes [64]. This could be a possible factor why Kirwan et al. [65] found Irish diets to be unsustainable when measuring data from the national adult nutrition survey against lifecycle assessment data on greenhouse gas emissions. To move towards more sustainable and healthy dietary habits, preferences, and cultures need to be considered [65]. Perceived value has been shown to have an impact on intention when analyzing the theory of planned behavior in relation to the adoption of sustainable consumption patterns, consumer effectiveness was also shown to impact behaviour [66,67]. However, it is suggested in this study that if one member of the household embraced change, they could bring their family along with them, like ***Anna aged 26–45 yrs*** discovered when her niece came to stay, *“my niece became vegetarian, for environmental reasons, and stayed with us for a month. I said, I’m not making another dinner because I have 2 fussy kids already, so we ate vegetarian for the whole month and now I’m building my vegetarian repertoire”*.

### 3.5. Facilitators and Barriers towards the Adoption of Sustainable Diets

**Figure 6:**
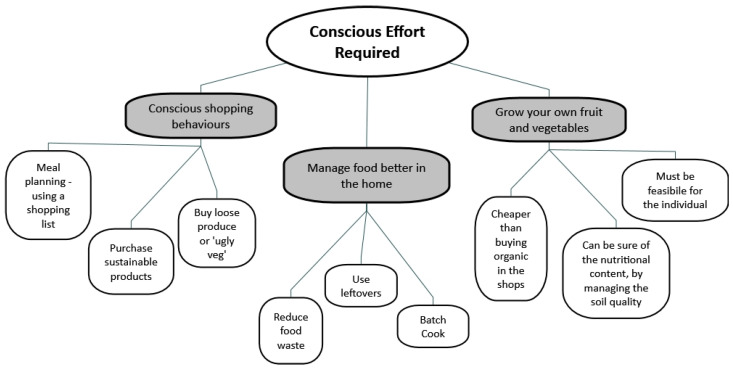
Map taken from NVivo, showing the main codes identified by the participants connected with the maintenance of a sustainable diet.

Participants believed that individual conscious effort was needed to maintain a sustainable diet, such as making a conscious effort when shopping, managing food better at home, and growing their own fruit and vegetables. Figure 6 illustrates how current habits could be adapted to improve sustainability. Similar subthemes were identified within the *‘changing habits’* theme mentioned earlier. However, the participants believed that the ability to change is sometimes outside of their control. 

Regardless of their good intentions to adopt more sustainable diets, several barriers exist (as seen in Supplementary Table S2). These barriers were often attributed to time constraints as the participants highlighted their busy lives. Other factors such as price and affordability were noted in numerous studies as a barrier to the adoption of sustainable diets [64,68]. This study suggested that, even though local products and organic products were viewed as sustainable food offerings, costs were often prohibitive. In contrast, a 2020 report from Bord Bia found that 47% of Irish Consumers are willing to pay more for organic foods, with 25% believing they are better for the environment [69]. Participants over 60 years of age in this study were driven by their values and sensory properties more than cost but did acknowledge that food is expensive and not everyone can afford to purchase healthy foods. This indicates that societal changes are required to ensure that sustainable consumption is moved from a ‘niche, novel’ status to more mainstream to allow all citizens to have the ability to improve their dietary practices and move towards more sustainable consumption. This is of critical importance due to the current ‘cost of living crisis’ with food prices and other goods rising rapidly [70]. According to the Central Statistics Office, Irish food prices in 2021 were 17% above the EU average [71], and fruits, vegetables, and potatoes were 14% above the EU average [54]. Yet, it was found in this study, that those who followed a vegetarian lifestyle stated their weekly grocery shopping costs were less than 80 EUR per week, suggesting a move to a more plant-based diet may help with spiralling food costs. 

However, participants in the current study acknowledged they had difficulty incorporating one or two vegetarian meals into their weekly routine, mostly due to sensorial factors, such as taste and flavour. This was also found in other studies [34,72,73] with participants focusing on the tastiness of meat in comparison to the alternatives. Market research in the UK noted that those who signed up for Veganuary in 2023 did not finish the monthly challenge, citing the higher cost of meat alternatives as a contributing factor [74]. However, the reluctance found in this study was mostly due to a lack of knowledge of vegetarian dishes, along with a trend of reverting to meat-based meals due to familiarity and the knowledge that they are a known source of protein. Hoek et al. [52] had similar results with Australian consumers who believed they needed the nutrients provided by meat and dairy products. The strong Irish farming culture where *‘you know where your products are coming from’* also encourages traditional dietary practices. The reinforcement of this mindset is of particular importance to the agri-food industry that is struggling with climate targets to reduce their greenhouse gas emissions (GHGEs) whilst attempting to maintain profitability. The Irish government has a roadmap to assist in ensuring the survival of the farming community by addressing sustainability factors and promoting the purchase of local produce, and this is a work in progress [41]. 

The impact of food choice motives on the current diet and the barriers, which impact potential dietary changes, highlight the many, almost involuntary trade-offs (code associated with the barriers in Supplementary Table S2) made by consumers on an ongoing basis. Time constraints in particular impact the decision-making process, with convenience and often the unhealthy, non-sustainable option being chosen, like the case of ***Louise, 26–45 yrs, step-mum to 3 kids,*** *stating “I would try to add protein, carbs, and veg but then the last few months it was it just was really busy. So, it was like frozen pizzas and takeaways and things like that”*.

Other factors related to knowing that certain foods are unsustainable due to air miles or excessive use of natural resources and choosing them anyway because of personal likes and dislikes, or other health-related factors such as beneficial nutritional content. *“I suppose we used to eat an awful lot of avocados. And we’ve kind of cut down on them. For that reason, like, even though I still love them. And if I was out and I saw an avocado on the menu, I’d probably eat it”, **Anna 26–45 yrs, lecturer.***

One of the most common barriers discussed by the participants was linked to affordability, the products they deemed sustainable such as organic or local were often outside their grasp due to their current situation. Like ***Dan 26–45 yrs, Postgraduate student in Nutrition,*** explained *“I feel like my diet is quite sustainable, being a research student, I don’t have a huge income. So, if I had a slightly larger income, it might be a bit more sustainable”*. This was also evident in insights from Bord Bia’s 2020 consumer report where fears of further recessions overshadowed the potential ability to consistently purchase environmentally friendly sustainable foods [69]. 

Trade-offs were also recognised in other studies that mention the external and internal factors linked to the consumers’ individual circumstances [64]. The results of this study show that the consumers are aware that changes are required at a personal and societal level; however, they did not believe that society was set up to encourage these changes, particularly with the fast pace of everyday life. This highlights the outside factors that are impacting or ‘believed’ to be impacting the participant’s adoption of more sustainable dietary practices as explained by the theory of planned behaviour [67].

*“We live in a fast world where we want to go out for dinner, we want to meet friends after work, we want to be on seven different tag rugby teams, or whatever so from a social point of view, I feel that’s the biggest factor in stopping people having sustainable diets.”*

***Nina, 26–45 yrs, Dietician.***

It was also acknowledged that convenience has become the norm resulting in the loss of ‘basic’ cooking skills to produce sustainable healthy meals. Moreover, time to batch cook sustainable foods is not available for all consumers, and these options in restaurants may be excessive in terms of cost, meaning non-sustainable alternatives may be chosen. Societal changes may also be required to assist consumers in adopting positive choices. 

It was recognised that a conscious effort would be required by consumers to make informed choices; moreover, for sustainable products to be successful, they would need to meet the key food choice motives identified, such as being sensory-acceptable, readily available, and affordable to encourage a sustained change and overcome potential detrimental trade-offs. 

### 3.6. Circular Eating

When addressing the second research question, which focused on the factors that affect the willingness to consume food products containing peels, trimmings, or by-products generated from the fruit and vegetable sector, it was important to gauge the consumer’s awareness of where food waste occurs. It was also important to understand their perception of the use of the parts of the fruit and vegetables that are traditionally discarded. 

#### 3.6.1. Where Food Waste Occurs and Why

The participants demonstrated a good understanding of the extent of food wasted in the homes. They also identified primary processing, food service, and retail as contributing to the problem, which is in contrast to other studies, which suggested consumers were unaware of food waste outside of their household [32]. 

Domestically, they believed the waste was generated between ‘the supermarket and the kitchen’ and suggested numerous ways to reduce the amount of food waste generated through better management in the home. The impact of food choice motives linked to consumer-related factors was also shown to have an impact on domestic food waste. This was situation-dependent due to busy lives, or often connected with having children in the home, with leftovers not used on time or products purchased for the children, not consumed within their use-by dates, and having to be discarded. The potential lack of understanding of expiration dates and the lack of awareness of when food is safe to consume or not were identified as another factor leading to unnecessary food waste. Other studies in the EU and US agreed with this finding and recommended changes in legislation and increased education in this area to make expiration dates clearer for the consumer [75,76]. Some of the participants felt that food waste, which goes for composting, should not be categorised as food waste indicating that there is also a lack of education about the food waste hierarchy.

At primary processing, the participants suggested a link between the products available to them in the supermarkets and the generation of food waste along this supply chain. Their understanding of this was linked to quality specifications and over-producing to meet orders. They did suggest climate change was impacting the harvesting of produce, but mostly it was down to retail requirements, which is confirmed in other studies [6,77,78]. However, the results of this study suggest the responsibility for household food waste generation is linked to the retailer and not the consumer alone; this was also found by Lemaire and Limbourg’s [1] review of European studies, who noted in-store promotions and incentives cause consumers to purchase more than they need. When discussing the concept of purchasing ‘ugly veg’, it was noted that there was a lack of availability of imperfect fruits or vegetables for sale in both supermarkets and farmers’ markets. Their food choice motives do suggest that quality in terms of freshness but also appearance drives their purchases; however, if the choice is not available, it is difficult to change this habit. 

This study found that the management of food waste in terms of disposal options available was limited to location and services in that area. For participants who live rurally, they had the option of composting at home. The food waste reduction targets to reach goals set by SDG 12.3, agreed at the EU and National levels had not filtered down to the consumers interviewed. They were sceptical and showed a level of distrust with the government regarding the ability to achieve these targets within the timeframe set and suggested that consumer education at home and retail level would be required to help achieve success. This demonstrates a potential lack of involvement on a societal level when tackling issues like food waste. Public discourse would be needed to show the extent of food waste present and the benefits of trying to reduce this problem through more sustainable practices, such as managing food better in the home as seen in Figure 5. It would also educate consumers on the benefits of using as much of this surplus material as possible in ways such as circular eating. Public discussions and media campaigns on food waste reduction in Denmark and Italy have been shown to increase the acceptance of waste-to-value foods [16,18]. The participants from this study also highlighted that the financial burden of disposing of food waste should help to encourage all stakeholders to manage this more effectively. Agreeing with the UN’s environmental program identified the potential to create cost savings and add value by managing waste streams more effectively [79]. 

#### 3.6.2. Acceptance of Circular Eating

During the interviews, the participants were asked ‘Have you heard of the term circular eating?’. Only two out of fifteen participants had come across the term previously, both were aged 18–25 years and had a background in nutritional or environmental science, and most participants in this study were older than 26 years of age. A study conducted with millennials in Italy found that 62% of their respondents had heard of foods made with upcycled ingredients [17], whereas a Danish study with the majority of participants older than 33 years of age found a lack of knowledge of upcycled foods [19], highlighting age as a potential factor in the awareness of circular eating. Another study comparing consumers in the US and China found low self-reported knowledge of upcycled foods in both countries (20% in the US and 30% in China) [80]. The participants were also asked ‘what is your understanding of this term?’. Those who had not heard of circular eating before referenced more mainstream terms such as ‘circular economy’ and ‘recycling’ when asked if they would like to guess what circular eating was based on the word itself. They were then informed that ‘during fruit and vegetable processing, trimmings, peel and other by-products usually end up in the waste stream’. They were asked ‘What do you think of these by-products being used as ingredients in a food?’ and ‘would you consume a product containing these ingredients’. Most participants (n = 12) were in favour of this practice with some conditions, such as the sensory appeal of the final food products and that the food industry should not profit at the expense of the consumer when developing and selling these products. The remaining participants thought the idea was fine *‘in theory’* but had major concerns specifically around food safety managed by the food industry. Overall, acceptance of waste-to-value products was affected by three factors: (1) Quality and Food Safety, (2) Subjective-Personal Preference, and (3) Economic factors, which follow the food choices of the participants (product and consumer-related factors). Table 4 shows the barriers and facilitators associated with factors one and two. The codes referenced most often are highlighted in bold. The Economic factors, such as concern about added processing costs, excessive profits by the companies at the expense of the consumer, and income-dependent access to the products, were mostly identified as potential barriers to acceptance and are not included in Table 4. To remove economic barriers like this, it would be critical to create a better dialogue between industry and consumers to explain the factors that may increase the cost of circular eating but are needed to ensure food safety [81,82]. 

Having little prior knowledge of circular eating, 80% of the respondents in this study viewed the concept positively. Particularly, as they suggested, there is an incredible loss of nutrients in the material that is discarded such as peel and skin of fruit and vegetables. This agrees with the findings of Spiker et al. [83] and Sager et al. [84] who mentioned nutrients such as water-soluble vitamins and fibres, which could be brought back into the food chain by using this material. This was found by Grasso et al. to be the driver, also for the consumption of upcycled foods by consumers in China [80]. Food safety was listed as an obstacle for some, due to the risk of pesticides present in the skin or peel of the fruit or vegetable getting into the food system, while the study conducted by Grasso et al. [80] found that some consumers in China consider upcycled “foods of higher quality that are safe to consume”. In the same study, only a small number of participants from the US agreed with that concept [80]. 

Food safety concern was also noted in other studies in terms of processing food waste material [85,86,87]. The EU regulations governing the use of pesticides are strictly adhered to, the promotion of this fact along with regular monitoring by food producers should go some way to alleviating the concerns of the consumers [88]. However, organic farming, which uses alternative methods to pesticides, may be viewed in a more favourable light by consumers. Bord Bia’s 2020 organic research report [69] confirmed a growth in the organic product sector with 47% of consumers willing to pay more for organic produce. The main reason for this was consumers suggesting ‘that it is better for your health’, However, local produce was preferred over organic [69]. It would be important to note that in this study, the cost of organic produce, regardless of being viewed as the more sustainable option, was noted as being expensive, and affordability was a major barrier within product-related food choice motives. The consumers interviewed in this study also had issues with the food industry profiting at the expense of the consumer by using material that would have previously gone into the waste stream.

Even though legislative frameworks such as waste management legislation and novel food legislation are in place to protect consumers, it is important to bring the consumer along the journey in terms of novel food production [16,85]. Improved communication on novel food and circular eating may work towards alleviating issues discovered in this study in terms of the trustworthiness of the food industry, based on prior bad publicity around the use of unhealthy ingredients. Such as transparent food labelling detailing the types of by-products used and the safety measures employed, or the reduction in food waste as a result of using this material, showcasing the sustainability factor associated with the product. Kasza et al. [85] noted the benefits not limited to waste reduction but also in terms of potential for job creation, which may help towards improving consumer acceptance. Aschemann-Witzel and Peschel [18] also agreed education and communication were key to encouraging positive changes. In following Coderoni and Perito’s [16] recommendations to educate the consumer on food processing challenges, there would be a means of creating some empathy with the industries’ need to improve their profitability by using by-products rather than discarding them, as explained by Lin et al. [89]. Garcia-Garcia et al. [82] confirmed there must be an end user for any waste-to-value products developed, so improving transparency and creating a clear, honest dialogue between the food industry and consumers is crucial to the acceptance of circular eating. Moshtaghian et al. [90] suggested adjusting the food waste hierarchy to include upcycled food production above animal feed, to encourage the industry to see this as a recognised option.

Transparency of information, particularly when promoting the environmental benefits of waste-to-value or upcycled products, is something that was suggested in this study and other research findings [19,90,91]. However, it was also noted by participants in the present study, that perhaps consumer information about the use of upcycled ingredients was unnecessary, as ‘*carrot peelings are still part of carrot*’. This was also discussed in a review paper by Aschemann-Witzel and Stangherlin [20] suggesting that ultimately not disclosing the origin of these types of ingredients could adversely impact the producer, as the consumer may reject the product if they discovered later that waste or surplus material was used. Aschemann-Witzel and Peschel [18] noted brand design would be important in the promotion of novel food, and this was also evident in this study, with participants stating that the promotion of sustainability factors could increase the uptake of circular eating and waste to value products, particularly if known brands were involved. Aschemann-Witzel et al. [91] recognizes the potential for circular food systems to contribute to reduced food waste and improved sustainability but, as this current study also confirmed, the concept needs promotion to avoid unnecessary confusion. Different strategies may be required for different market segments as Zhang et al. [92] found Generation X consumers (born 1965–1979) were more concerned about potential food safety issues with upcycled foods. Food safety issues were also suggested in this study as something to address before full acceptance of circular eating, and the participants who suggested this were also in Generation X or older age categories. 

Like the adoption of sustainable diets, food choice motive ‘consumer-related factors’ such as personal likes and dislikes have an impact on the acceptance of waste-to-value food or upcycled food products. The sensorial factors were discussed by those interviewed, stating the product must “look and taste good” if it is to be accepted. Sensorial factors were also mentioned by Moshtaghian et al. [90] and Aschemann-Witzel and Peschel [18]. In the latter’s study, pilot trials indicated the consumers anticipated a poor taste from plant-based ingredients and were reluctant to try new upcycled products. This was overcome by the addition of familiar well-liked flavours such as chocolate, which improved uptake in the main trial [18]. To avoid any food disgust or neophobia, it could be beneficial to address negative organoleptic issues by using known flavours and by ensuring the products deliver on taste. Neophobia, although mentioned by one participant, was not found to be significant in this study, though it was evident in other studies and should not be discounted [16].

### 3.7. Sources of Consumer Education/Information 

There was a lack of education about what makes food or ingredients sustainable. The participants showed awareness of environmental issues associated with sustainability and which foods production are particularly detrimental to the planet’s natural resources. This knowledge has mostly filtered down from mainstream media or the internet. Similar to findings from Chandon et al. [93] and Hieke et al. [94], the current study found that participants with science and nutrition education made more informed decisions by getting their dietary information from reputable sources, but they also struggled to define a sustainable diet when asked during the interviews. To create positive changes in dietary habits, education is required across the population [33]. As found by Larson et al. [95], if positive values in sustainable consumption are present from adolescence into adulthood, this would lead to improved dietary practices for life. The importance of education was also recognized in the awareness of the food waste problem. Creating an awareness can empower consumers to better manage food waste in the home, and in turn, be more agreeable to new concepts such as circular eating [16,18]. Greater awareness would start a national discussion on food waste and potentially remove some of the stigma associated with its potential use in upcycled food products, particularly if avoidable food waste or surplus material is brought back into the food supply chain to tackle issues such as food poverty. In terms of the consumers’ contribution to food waste generated at production, processing, and retail levels, the knowledge divided between how food is produced and food that is available in the retailers was evident in this study, as was the lack of trust in the food industry. Coderoni and Perito [16] suggested bringing the consumer on the development journey to educate them on the product. The same concept was also suggested in this study, educating the consumer on the benefits of circular eating via food labeling would possibly result in greater acceptance and potentially a greater understanding between the industry and the consumer.

Knowing the consumers’ food choice motives can help to identify new ways to encourage positive change. Education and public discourse are major factors in facilitating positive change, as attitude towards and intention to change needs to be encouraged. 

This study highlighted the numerous food choice motives that have the potential to influence the adoption of sustainable diets and sustainable practices associated with food like circular eating. There are many barriers to be overcome for a conscious dietary shift to occur, but it was evident from those interviewed that the willingness is there to make these changes. However, assistance is required at a societal level to aid consumers in making positive, sustainable choices. In terms of circular eating, although a very new concept to most, it was viewed as a valuable way to bring nutrients back into the food supply chain, reduce unnecessary waste, and encourage sustainable production and consumption patterns.

### 3.8. Limitations

Prior to discussing the conclusions, it is important to note some limitations of this study. The generation of the data was based on the interviews of fifteen participants, which could be considered a small number to make generalizations for the findings. However, the interviews provided rich data that helped describe the perception of sustainable diets and their connection with the acceptance of circular eating. Due to the recruitment process based on gatekeepers, word of mouth, and snowball sampling most of the participants interviewed expressed an interest in new food concepts and sustainability. This could potentially skew the results as it does not capture the voices of those who are not interested in the topic. Also, all but one of the participants were educated to a degree level or post-graduate qualification. It has been shown that those educated to a higher level are also more open to the adoption of sustainable dietary practices [34]. However, by using the sample triangulation as seen in Figure 2, it was ensured that the voice of a participant with second-level education was included in the data set.

## 4. Conclusions

The aim of this study was to understand how Irish consumers perceive sustainable diets and what factors influence their willingness to consume food products containing peels, trimmings, or by-products generated during fruit and vegetable processing. The qualitative data generated by this study gave a rich understanding of the trade-offs made by those interviewed in choosing the foods they purchase and how they manage food within their own micro-environment. Their food choice motives heavily influenced them and should be considered when identifying ways of encouraging the adoption of sustainable diets. The results showed an awareness of environmental degradation due to the food production system, filtering down to the consumer via media and news streams, but there needs to be greater communication describing sustainability, and sustainable diets, and breaking it down further to what foods are sustainable and why. Sustainability needs to be a user-friendly term with transparent information derived from reputable sources avoiding over-exposure and ‘green washing’. Educating consumers on how to make small changes in their diet to benefit their health and the environment will help to create new sustainable habits. 

The results from this study reflected positively on the concept of circular eating as a proactive way of reducing food waste in the horticulture sector. The participants recognized the lost nutrients present in the peels and trimmings of fruits and vegetables, which should be brought back into the food stream. They did expect action and transparency from the food industry in terms of the safe management of surplus material and ensuring consumers were not overly charged when it came to purchasing these products. The promotion of the environmental benefits of using these ingredients rather than wasting them was viewed as important by the participants, and food labelling should reflect this. Changes in public policy messaging, and utilising more online forums, which were confirmed as the most frequent methods of accessing dietary information in this study, could inform consumers about the impact of food waste decaying in landfill and the generation of greenhouse gas emissions. This could help create a link for the consumer between sustainable practices, food waste reduction, and circular eating. Future research should focus on exploring how this messaging can impact the acceptance of products containing surplus material. 

After gaining an insight into consumers’ perception of sustainable diets and their views on the concept of circular eating, there was an obvious level of distrust present with regard to the food industry. To address this, it would be essential to explore the managing of surplus material from an industry stakeholders’ perspective. It would also be important to bring the consumer’s voice into the development process to ensure that a circular eating market exists, avoiding the generation of further waste material. This study highlighted the opinion of consumers mostly educated to a degree or post-graduate level; it would be also beneficial to expand this research to capture participants with different educational backgrounds. Finally, this qualitative study could guide the development of a survey for the collection of quantitative data from a larger representative sample of the Irish population.

## Figures and Tables

**Table 1:** Sociodemographic characteristics of the participants interviewed.

Sociodemographic Characteristics		Frequency (n)
Gender	Male	6
	Female	9
Age	18–25 years	2
	26–45 years	9
	46–65 years	3
	65+ years	1
Education level	Second Level	1
	Third Level	7
	Post Grad	7
No. of people in the household	1	3
	2	3
	3	4
	4	5
Responsibility for the main weekly grocery shop	Sole	7
	Shared	7
	Parent	1
Money spent on weekly grocery shop	€80–€100	5
	€101–€150	7
	€151–€200	1
	€200+	1
	Don’t know	1
Area lived in	Rural	11
	Urban	4
Preferred diet	Meat eater	11
	Vegetarian	2
	Vegan	2

**Table 2:** Main themes generated from the interviews and frequency they were mentioned and the associated subthemes.

Themes	Frequency (n)	Subthemes
Sustainable Diets	801	Consumers’ awareness of sustainability Consumers’ beliefs regarding sustainable foods How to maintain a sustainable diet Limitations to adopting a sustainable diet
Food Choice Motives *	590	**Consumer-related factors** **Product-related factors** **Sustainability factors**
Awareness of Food Waste *	314	**Food waste management** **Food waste causes** **Where food waste occurs**
Sources of Consumer Education	118	Online Sources Retail-led Mainstream Media Community-led Wellness Industry Advertising campaigns Educational Background Peers/Friends Influence
Acceptance of Circular Eating	95	Waste-to-value (WTV) product acceptance Labelling of WTV ingredients
Changing Habits	55	Eating behaviours Shopping behaviours Sustainability factors Household engagement Change is challenging

* Overarching themes are shown in **bold** in the table above.

**Table 3:** Food choice motives’ theme—Breakdown of subthemes and associated codes including the frequency (n) these were mentioned.

Subthemes (References (n))	Associated Codes (References (n))
Consumer-related factors (246)	Health considerations (106) Eating Habits (64) The micro-environment (39) Socio-demographic factors (19) Environmental concerns and beliefs (9)
Product-related factors (246)	Quality factors (122) Economic factors (66) Convenience (25)
Sustainability factors (98)	Environmental issues (78) Social issues (20)

**Table 4:** Codes associated with the subtheme waste-to-value product acceptance, broken down into potential barriers or facilitators impacting acceptance.

Codes	Barriers to Acceptance	Facilitators for Acceptance
Quality and Food Safety	**Food safety concerns (12) ^1^**	**Clear information on packs (7)**
	Difficulty trusting the industry (3)	Awareness of lost nutrients (3)
Subjective—Personal Preference	Personal likes and dislikes (7)	**Sensory appeal (7)**
	**People may struggle with the concept (8)**	Branding influence (2)

^1^ Frequencies are listed in brackets beside each code, codes with higher frequencies per group of barriers and facilitators are shown in **bold.**

## Data Availability

The data presented in this study are available on request from the corresponding author. The data is not publicly available due to ethical reasons.

## Supplementary Materials

The following supporting information can be downloaded at: https://www.mdpi.com/article/10.3390/foods12214003/s1, Section S1: Main Research Questions and Embedded Questions; Table S1a: Question Grid for Interviews Sociodemographic Information; Table S1b: Question Grid for Interviews; Section S2: Example of NVivo visualization tools to assist when conducting Reflexive Thematic Analysis using NVivo to manage the data; Figure S1: Screenshot of memo used in NVivo to help visualise data; Figure S2: Screenshot of annotations used to explain coding during data analysis; Figure S3: Example of a concept map from NVivo to help with visualization of data during reflexive thematic analysis; Figure S4: Example of a flow chart from NVivo for visualization of data when conducting Reflexive Thematic Analysis; Table S2: Codes and quotes associated with the subtheme of barriers identified by the participants which may prevent the adoption of a sustainable diet.
