# Supplementary material for: Current Perceptions of Sustainable Diets in Ireland and the Outlook of Circular Eating Practices
Source: Foods. 2023 Nov 1;12(21):4003. doi: 10.3390/foods12214003 (PMC10648305; doi:10.3390/foods12214003)
Supplement: Supplementary file 1 [file foods-12-04003-s001.zip › foods-2677924-supplementary.pdf]

Supplementary Material

Section S1 Interview Question Grid

AIM: To investigate the perception of sustainable diets among Irish consumers and to explore what factors affect their willingness to consume food products containing peels and trimmings generated during fruit and vegetable processing.

Research question 1: How do Irish consumers perceive sustainable diets?

Embedded question (EQ) 1a: What are the factors related to the sociodemographic characteristics of participants?

Embedded question (EQ) 1b: What are the characteristics of the diets that make them sustainable according to the participants?

Embedded question (EQ) 1c: How do the dietary behaviors of the participants match with the FAO's definition of a sustainable diet?

Research question 2: Which factors affect the willingness to consume food products containing peels, trimmings or by-products generated during fruit and vegetable processing?

Embedded question (EQ) 2a: How do consumers currently handle food waste at home?

Embedded question (EQ) 2b: How do consumers perceive the food products containing peels, trimmings or by-products generated during fruit and vegetable processing?

Table S1a: Question Grid for Interviews – Sociodemographic Information.

Topic	Question	Option	Reference/Rationale
**Demographic Information**	1.What is your gender?<br><br>2.What is your age?<br><br>3.Please select your highest level of education?<br>4.Please select your current employment status?<br><br>5.What is your financial status /average income over the last 3 years?<br><br>6.How many live in your household?	Male, Female, prefer not to say.<br><br>18- 25; 26-45; 46-65; 65+<br><br>Junior Cert, Leaving Cert, Degree, Post Graduate Degree, PhD.<br><br>Unemployed, Part-time employment, Full-time employment, Self-employed, Work in the Home, Student.<br><br>Low (inadequate to cover daily expenses), Moderate (trying hard to cover daily expenses), Good (adequately covering daily expenses),<br>Very Good (easily covers daily expenses).<br><br>Selection:[1, 2, 3, 4, 5+]	***These questions are asked to answer the EQ1a. They will be presented in a form of a survey before the interview.***<br><br>Aschemann-Witzel *et al.*, [18] discovered gender interaction, noting that females were more likely to be in favour of plant-based products whilst males were more open to novel products or novel processing techniques.<br><br>Culliford and Bradbury [34] showed an interaction between gender and age in the readiness to adopt sustainable dietary behaviours.<br>Barone *et al.*, [33] showed an association between age, education and where respondents live in terms of knowledge around sustainable diets.<br><br>Mirabella *et al.*, [81] noted that modification of waste streams to ensure food safety may be costly and a high value-added product would be needed to justify the expense. It is important to understand the average income and weekly spend of consumers to ascertain if a more costly food product would be a viable option.

	6.1. How many under the age of 18?<br>7.Responsibility for grocery shopping<br><br>7.1. What does your average weekly spend on grocery shopping look like?<br><br>8.Where do you live?	Selection: Relevant age brackets [0-2yrs, 3-6yrs, 7-12yrs, 13-17yrs]<br>Selection:<br>[myself, parent, shared responsibility, partner]<br><br>Selection: [€80-€100, €101-€150, €151-€200, €200+]<br><br>Selection: [Rural - countryside, Rural - seaside, Urban – small town, Urban – large city.]	

Table S1b: Question Grid for Interviews.

Sustainable diets	
Interview Questions	***Reference/Rationale***
1. What factors are important to you and your family in terms of the food you purchase in your weekly grocery shop? Suggested Prompts if required [healthiness, environmentally friendly, animal friendly, convenience, tastes good, good price, familiar, Weight management, looks appealing, fits in with my religion/culture, caters to my family needs]	***Answering EQ 1c*** *FAO* 2010 [44] have defined what sustainable diets should be, it is important to identify what Irish consumers believe is important in relation to their diet, to see how this reflects against the suggested sustainable diet. Hoek *et al.,* [52] looked at the emotions, perceptions and attitudes that influence food behaviors by using a similar list of attributes, derived from Steptoe, Pollard & Wardle [27]. These questions will help us better understand the tradeoffs consumers make when choosing the food that they purchase.
2. Where would you look for information around food and your diet? Selection of Prompts if needed [Internet; TV/Radio/Podcasts; Food Labels; Science and Health Professionals; Magazines/Newspapers/Online Versions; Friends; Others (Training, lectures, conferences)]	***Answering EQ 1b*** Barone *et al.,* [33] uses qualitative data to assess Brazilian consumers attitude to sustainable diets and concluded that education was a necessity to inform consumer opinion. Therefore, knowing where the consumers get their information in relation to food and diet could help guide future education practices to evaluate if information from scientific and commercial sources are frequently used and how these impact eating motives, need for understanding and ability to evaluate information. Hieke *et al.,* [94] discovered those interested in scientific information/nutritional information are more likely to make positive dietary choices, rather than those focused on commercial information. It would be important to correlate the response of the Irish consumer to see if similar patterns exist.
3. Do you look at the labels when you buy food? *3a. What are you looking for on the labels?*	***Answering EQ 1b*** Aschemann-Witzel and Peschel [18]asked the question 'How circular will you eat?', using the addition of novel proteins in a chocolate plant-based drink, they researched consumer attitude. The information displayed on the packs they presented to participants showed a positive correlation to choose and impact on the environment. It is important to see if Irish consumers view food labels similarly.

	Hieke *et al.*, [94] found that information sourced scientifically and from nutritional information on packs positively correlated to healthy food choices. Positive responses to these questions would assist us in recommending how to display information on sustainable ingredients/products on the packaging.
**4. Can you talk me through the types of products consumed in your house on an average day?**<br>**5. What does your regular dinner/main meal look like?**<br>**6. How often would you eat meat / fish per week?**<br>6a. Why do you consume these in the mentioned frequency?	***These questions are answering EQ 1c, to understand how sustainable, the diets of the participants are.***<br>Questions linked to meat consumption are asked based on the suggestion by Willet *et al.*,[49] that a sustainable diet needs to reduce meat/fish intake to once or twice weekly. Asking the reason, they consume what they consume will also give insight into the factors affecting their food choice, which further support their answers from Q1
7. ***What comes to mind when you hear the term 'sustainability'?***<br>*8.* ***Could you please complete this sentence A sustainable diet is….***<br>***9. Can you name some characteristics of sustainable foods?***<br>***10. Can you name some characteristics of non-sustainable foods?***<br>*Prompts if required [Protective of biodiversity, Safe and healthy, Plant based, culturally acceptable, Affordable, Nutritionally Adequate, Low environmental impact, Optimize Natural Resources, Organic]*	***These questions will attempt to answer the EQ 1b,*** which is exploring what Irish consumers consider sustainable diet. The questions used here are based on the ones used by Barone *et al.*, [33].<br>An important element of the current research is to identify the Irish consumers' awareness of sustainability to identify if requirements as identified in the study of Barone *et al.*, [33] are needed, understanding where they currently get their information from will be important in terms of explaining their current behaviors in terms of diets.<br>Van Loo *et al.*,'s [43] study showed a clear relationship between attitude and behavior, with a strong association between healthy and sustainable eating and a plant-based diet. It is important to understand the Irish consumers perception of this.
11. **Have you come across the term 'sustainable development goals?**<br>*Prompt [If yes what is your understanding of this term?]*	***Answering EQ 1b***, trying to see if Irish consumers are aware of the term and if there is a connection between sustainable diets and sustainable development goals. "Achieving the sustainable development goals (SDGs)through sustainable food systems" *Sustainable and healthy diets for*

	the achieving the SDGs, the role of the consumer (Cecilia Rocha, Daniela Spagnuolo, pg. 143) It is important to understand the Irish consumers' awareness of SDGs
12. **How sustainable would you consider your diet?**<br><br>13. **How easy or difficult is it to follow a sustainable diet?** [Education, Price, Availability, Current Habits, Brand Loyalty, Cultural or Religious Impacts]	***Answering the EQ 1b and EQ 1c***<br>*FAO* 2010 *and Willet et al* 2019, define sustainable diets, based on the answers to Q1, 4 and 5 and their response to Q 12, we can easily see if the Irish consumers have a good understanding of sustainable diets.<br>Johnson *et al.*, [64] believes economic impact is the biggest barrier to changing to a sustainable diet, it would be good to understand the Irish consumers beliefs in relation to barriers.
Food waste	
Interview questions	***Reference/Rationale***
14. **What would you consider food waste?**	***This question will attempt to answer EQ2a***<br>Papargyropoulou *et al.*, [6] research focused on differentiating between food surplus and food waste, using the waste hierarchy as a guidance tool. It is important to this research to understand what Irish consumers consider as food waste.
15. **How do you currently manage your household food waste (*for example fruit and vegetable peelings, food not consumed etc.*)?**<br><br>Prompt if needed [general waste bin, compost bin collected by waste disposal company, compost bin in own garden, feed to animals, freeze and consume at another time, use in other food products/or smaller meals]	***This question will attempt to answer EQ2a***<br>It is important to understand how consumers currently view and handle their own household waste. Flanagan and Priyadarshini [5] researched Irish consumers attitude to food waste and its impact on global warming, it would be relevant in this study to compare their habits to food waste on sustainability, as attitude to waste may affect their willingness to consume product made from food that usually goes to a waste stream.
The Irish Government aim to halve our food waste by 2030.<br>16. ***How achievable do you think this target is?***<br>***15.a Why do you think we waste should be reduced?***	***This question will attempt to answer EQ2a***<br>Ireland has a Waste Action Plan for a circular economy, Ireland's National Waste Policy 2020-2050 . They aim to halve our food waste by

Prompt: [if not how do you think they should raise awareness?]<br>Prompts if required [biodiversity; water consumption; greenhouse gas emissions; Nitrogen & Phosphorus levels in soil]	2030, this is in line with the UN's sustainable development goal 12.3. Other policy documents include the Waste Framework Directive (2018/851/EC) and the Farm to Fork Strategy.<br>The EAT lancet report, Willet *et al.*, [49] states that reducing food loss/waste is a key factor across all proposed measures to ensure healthy and sustainable food systems for now and future generations, if the target of 50% reduction is achieved it has positive impacts on all aspects. The responses to these questions will relate to the Irish consumers' understanding of a link between sustainability and reducing food waste.
**17. How important is the appearance of the fruit and vegetables you purchase at the supermarket?**<br><br>Prompts if required: [Very Important, Important, Somewhat Important, Not Important] *(could include an image with this question so they have a visual)*	***These questions attempt to answer EQ2b***<br>Beausang *et al.*,[77] and FAO 2011 [4] mention the fact that retailers have cosmetic criteria assigned as a QA standard when purchasing from producers, this is driven by consumer expectation. I believe it is important to establish Irish consumers perception of this as if they do not want to purchase ugly vegetables for example this may corelate with their purchase intent of food ingredients/products containing peels, trimmings or by-products from fruit/vegetable processing industries.
During vegetable and fruit processing trimmings, peel and other by-products end up in waste streams.<br>**18. What do you think of these being used as ingredients in a food?**<br><br>**19. Would you consume a product containing these? Why? Why not?**<br><br>**20. How much would you pay for these products? Compared to products which do not contain these ingredients?**<br>Prompt if needed – more of less than usual pricing.<br><br>**21. How would you characterize/ describe these ingredients?**	***These questions attempt to answer EQ2b***<br>Maina *et al.,* [15], Mirabella *et al.,* [81] look at the potential for food waste in a circular economy.<br><br>Coderoni and Perito [16] reviewed the socio demographic and psychological features that rule the extent of which consumers purchase waste to value food and the circular economy. They concluded that the uptake of waste to value foods depends on consumers purchase choices and consumer acceptance is decisive in the development of successful novel foods. Its critical to understand the Irish consumers perception of this and their reasoning behind their purchase choices.<br><br>Mirabella *et al.,* [81] noted that modification of waste streams to ensure food safety may be costly and a high value-added product would be needed to justify the expense. It is important to understand the average

	income and weekly spend of consumers to ascertain if a more costly food product would be a viable option.
**22. Have you heard the term circular eating?**<br>**22.a. What is your understanding of this term?**	***These questions attempt to answer EQ2b as the circular eating references using all the material, reducing waste and closing the loop.***<br>Aschemann-Witzel and Peschel [1] asked the question 'How circular will you eat?', using the addition of novel proteins in a chocolate plant-based drink, they researched consumer attitude to this concept. Irish consumers awareness to the term and concept would correlate to their overall acceptance of sustainable food choices.

Section S2 Examples of visualization tools to assist when conducting Reflexive Thematic Analysis using NVivo for data management .

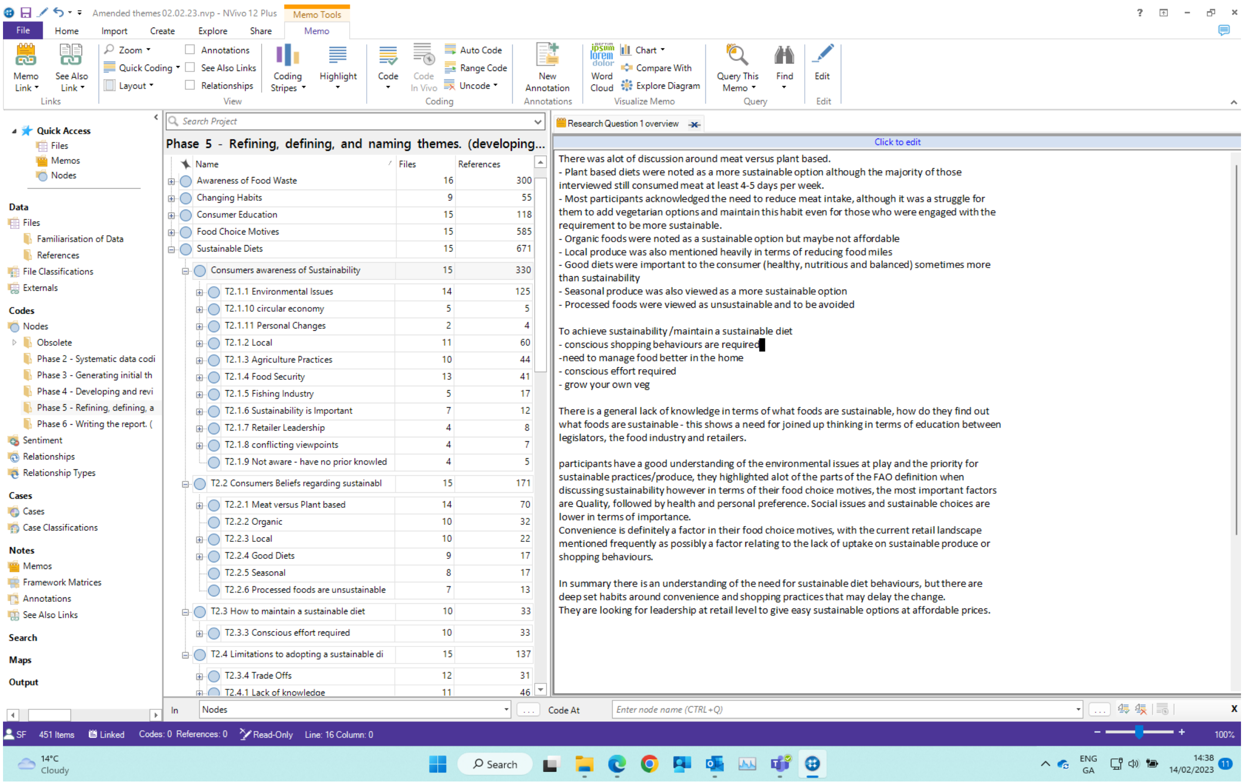

Figure S1: Screenshot of a memo used in NVivo to help visualize data, this example is focused on the first research question and highlights the discussion around meat versus plant based in the overall discussion on sustainable diets.

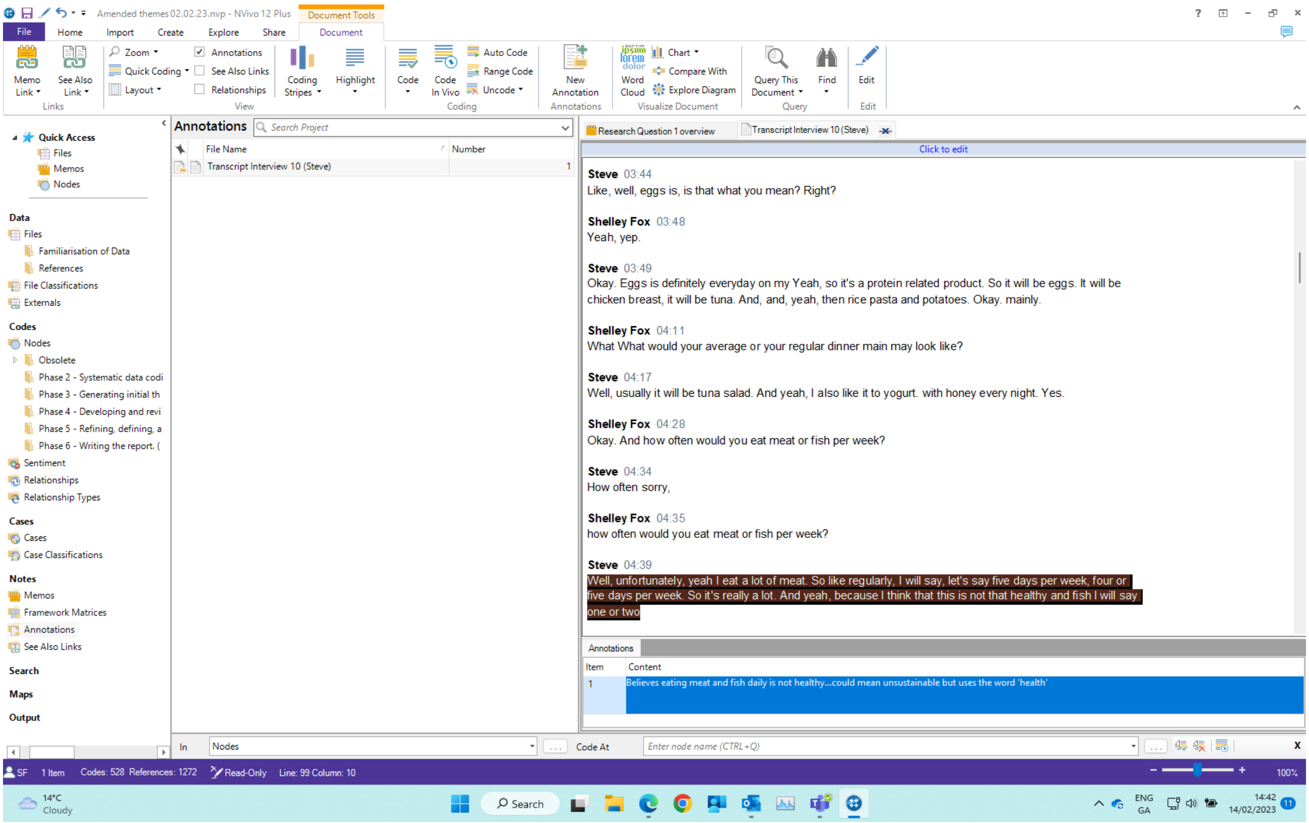

Figure S2: Screenshot of annotations used to explain coding during data analysis, to help explain the coding process, showing how the participant Steve (a pseudonym) believes consuming meat and fish daily as unhealthy.

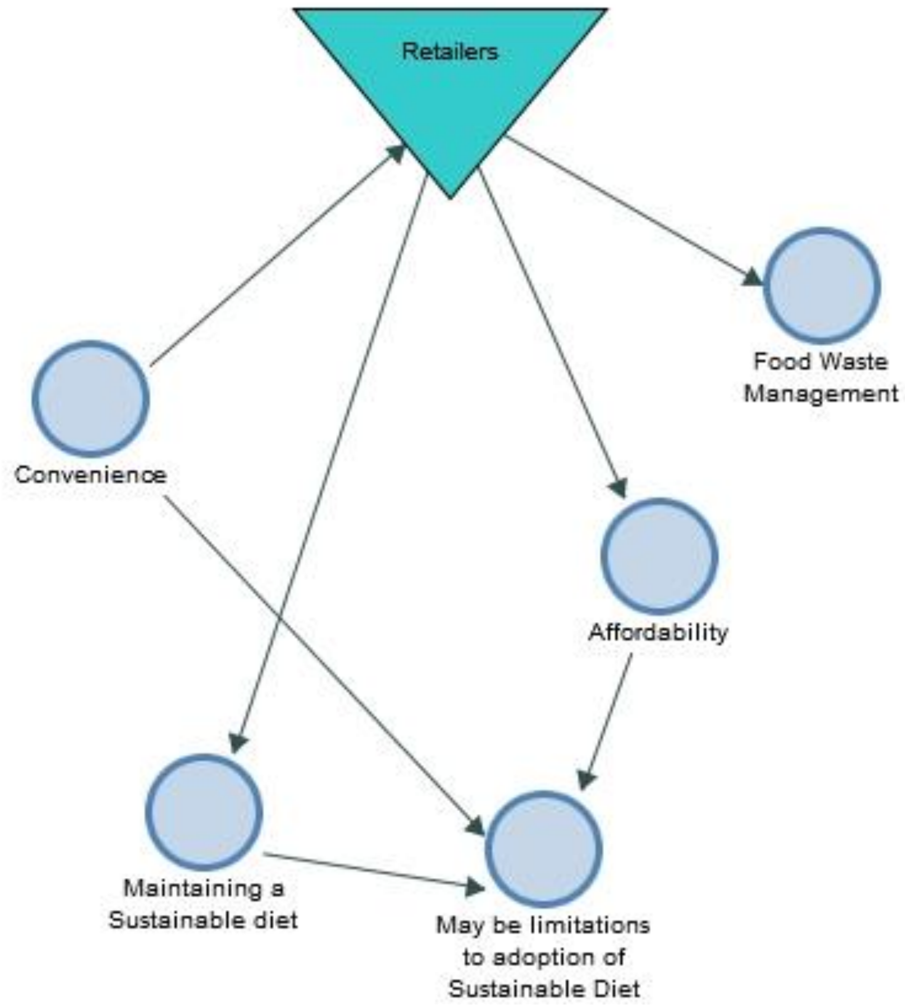

Figure S3: Example of a concept map from NVivo to help with visualisation of data during reflexive thematic analysis, this example highlights the connection between the retailers, sustainable diets and food waste management.

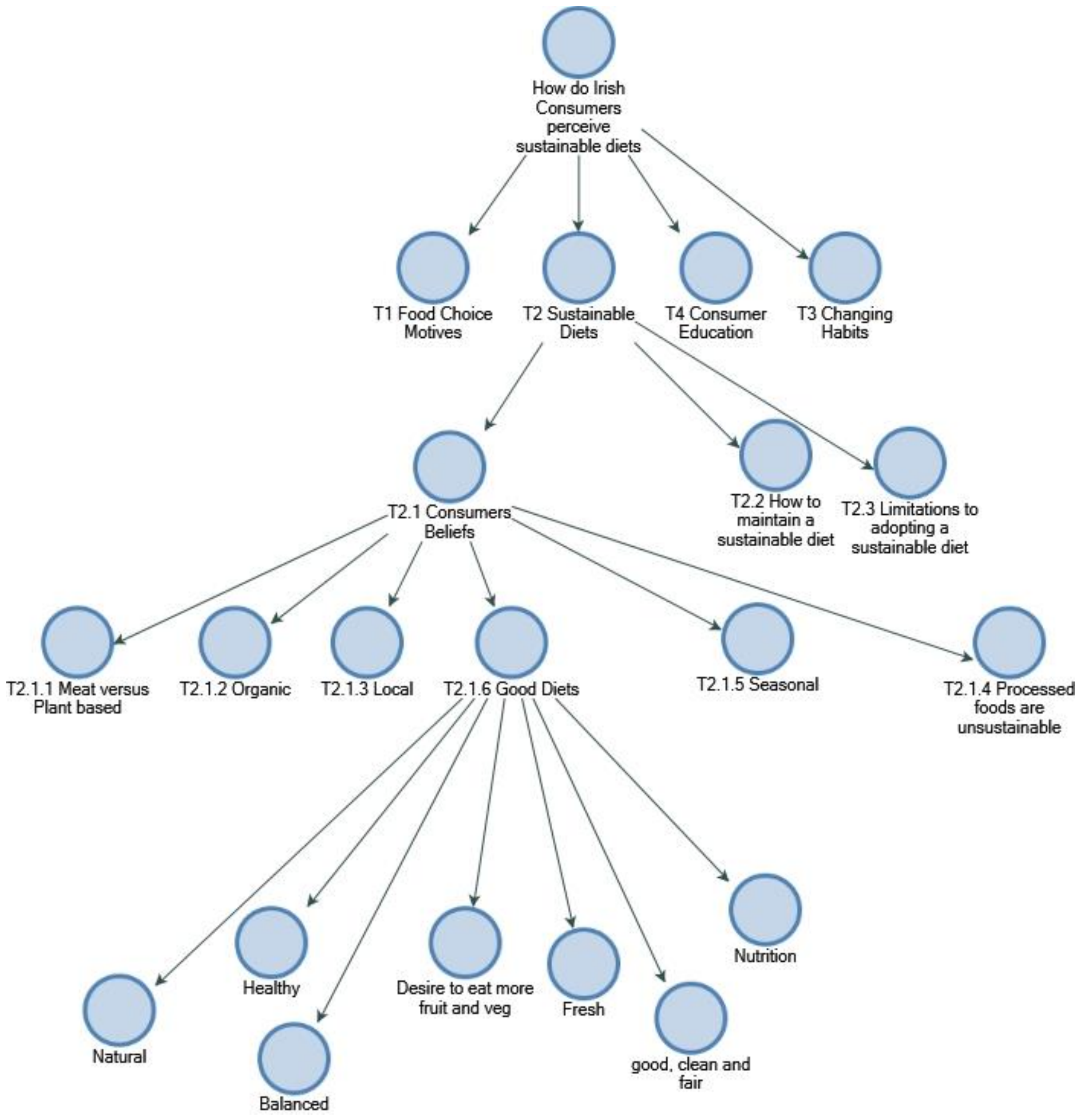

Figure S4: Example of a flow chart from NVivo for visualisation of data when conducting Reflexive Thematic Analysis, this follows the first research question 'How do Irish consumers perceive sustainable diets', shows the themes, and the subthemes and associated codes for the sustainable diet theme.

Table S2: Codes and quotes associated with the subtheme of barriers identified by the participants which may prevent the adoption of a sustainable diet .

Associated Codes	Quotes
Lack of knowledge	*"It doesn't come naturally to include veggie food".* *"You think nuts seeds are sustainable, I don't know where they come from. So, I don't know whether they're sustainable or not"*
Trade offs	*"If I had a slightly larger income, it might be a bit more in terms of sustainable".* *"We could shop locally and try to buy or like, but at the same time, if you want to buy avocados, you can't buy avocados that are grown down in Donegal"*
Difficult to Maintain	*"I went completely vegan. That was quite short lived and was on/off. And then vegetarian, but like I said, before, I've been eating bits of meat now over those last few weeks."* *"I think it is extremely difficult in today's society, because we don't have the time to bulk cook and change our recipes to be seasonal"*
Expensive	*"I tried to be to get organic and stuff, but also, price wise, I have to be realistic that sometimes I have to go for the cheaper option"*
Situation Dependent	*"If you're with a group of people like if everybody in my household, were more interested, it would probably make it easier".* *"I figured it is as sustainable as it could be, I could maybe if I had a higher income, I would consume better quality products"*
